# Supporting Post-ICU Recovery: A Narrative Review for General Practitioners

**DOI:** 10.3390/diseases13060183

**Published:** 2025-06-11

**Authors:** Charikleia S. Vrettou, Athina G. Mantelou

**Affiliations:** First Department of Critical Care Medicine & Pulmonary Services, School of Medicine, National and Kapodistrian University of Athens, “Evangelismos” Hospital, 106 76 Athens, Greece; athina.mantelou@gmail.com

**Keywords:** post-ICU syndrome, general practitioners, rehabilitation, critical illness

## Abstract

Survivors of intensive care unit (ICU) hospitalization often face persistent health challenges after discharge, collectively referred to as post-intensive care syndrome (PICS). This condition affects physical, cognitive, and mental health, significantly impacting patients’ quality of life and increasing their healthcare utilization. Additionally, caregivers may develop PICS-F (PICS family), experiencing stress-related health burdens. Despite the growing awareness of these issues, structured post-ICU follow-up remains inconsistent, leaving a gap in care that general practitioners (GPs) must often fill. This review examines the role of GPs in managing post-ICU patients, outlining common complications, screening tools, rehabilitation strategies, and potential areas for improved collaboration between primary care and ICU teams. Emphasizing a multidisciplinary and proactive approach, we propose practical interventions that GPs can adopt to enhance long-term recovery outcomes for ICU survivors.

## 1. Introduction

The reasons for intensive care unit (ICU) admission are diverse, encompassing a wide spectrum of medical conditions. Patients may require intensive care due to postoperative monitoring following major surgeries, including cardiac, thoracic, extensive abdominal, and neurosurgical procedures. Additionally, ICU admission is often necessitated by life-threatening conditions, such as respiratory failure, acute respiratory distress syndrome (ARDS), shock, and coma resulting from trauma or primary central nervous system injury [1,2,3,4]. The increasing utilization of ICUs reflects advancements in medical care and a growing population of patients with complex, severe illnesses [5,6,7]. ICUs provide highly specialized care, including continuous monitoring, multifactorial assessment, and life-support interventions. These interventions involve mechanical ventilation, renal replacement therapy, pharmacologic and mechanical circulatory support, and other advanced technologies, such as extracorporeal membrane oxygenation (ECMO) aimed at stabilizing critically ill patients [8,9,10]. While advances in medical knowledge and technology have significantly improved survival rates among ICU patients, they have also introduced new concerns regarding the long-term health and well-being of survivors [11,12].

**Figure 1:**
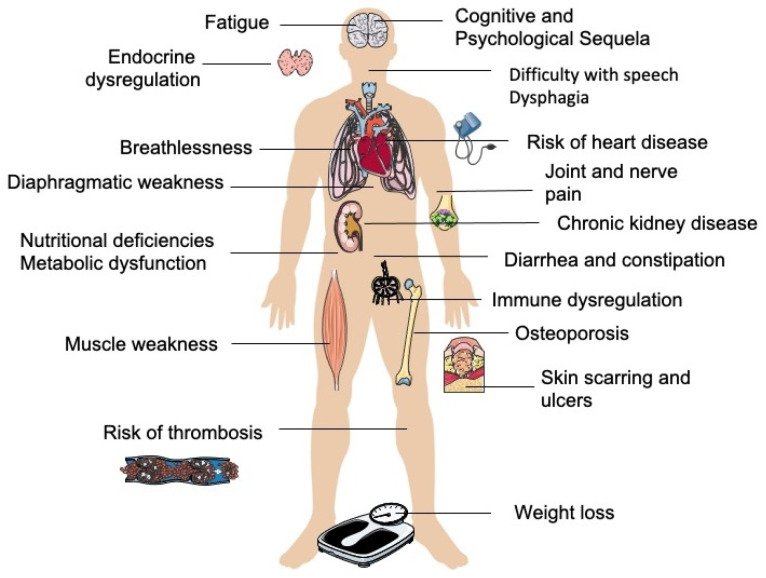
Multisystem complications associated with post-intensive care syndrome (PICS). Elements adapted from Servier Medical Art (CC BY 3.0, smart.servier.com).

The aftermath of critical illness can be profound, affecting physical, cognitive, and psychological functions, not only in patients but also in their caregivers [13,14,15,16,17]. Over the past decade, post-intensive care syndrome (PICS) has been increasingly recognized as a complex condition affecting ICU survivors. PICS manifests in the following three primary domains: 1. Physical impairments, such as muscle weakness, fatigue, chronic pain, and respiratory dysfunction. 2. Cognitive dysfunction, including memory impairment, decreased concentration, and executive dysfunction. 3. Psychological disorders, such as depression, anxiety, post-traumatic stress disorder (PTSD), and emotional distress [18,19]. Additionally, family members and caregivers of ICU survivors often experience significant psychological distress, leading to what is termed PICS-F (PICS family). Symptoms of PICS-F include depression, anxiety, PTSD, caregiver burnout, and chronic stress [19,20,21]. Table 1 and Figure 1 present the most frequently reported health issues associated with PICS.

The definition of PICS is crucial in shaping healthcare frameworks and guiding post-ICU care. However, its clinical diagnosis presents several challenges. These include 1. subjective symptomatology: many PICS-related symptoms, such as dyspnea and fatigue, are difficult to quantify objectively, making standardized diagnosis challenging. 2. Exclusion of neurological ICU patients: while patients with primary neurological injuries (e.g., traumatic brain injury, stroke, or central nervous system infections) frequently suffer from mobility restrictions and cognitive deficits, they are not traditionally included under PICS definitions due to the pre-existing central nervous system involvement. 3. Lack of standardized assessment tools: various studies use different scales and tools to evaluate PICS, leading to inconsistent prevalence estimates and making it difficult to compare outcomes across studies. 4. Evolving definition of PICS: an extended definition of PICS has been proposed to incorporate a wider range of post-ICU complications not included in the original framework. These include cardiovascular, metabolic, and immunological complications, which may emerge months or years after ICU discharge [22,23,24,25,26]. 5. Determining whether new or worsening symptoms are directly attributable to ICU care remains a challenge, particularly in older or multimorbid patients. While neurological and psychological complications are often discussed in the context of post-intensive care syndrome (PICS), many other chronic conditions—such as cardiovascular disease, kidney dysfunction, or diabetes—may also emerge or worsen following critical illness. However, these conditions are often difficult to disentangle from pre-existing comorbidities, complicating both diagnosis and long-term management [27]. Given the increasing reliance on general practitioners to oversee the long-term recovery of ICU survivors—often in the absence of structured follow-up services—this narrative review aims to synthesize current knowledge on post-ICU sequelae and highlight practical considerations for GPs tasked with managing this complex patient population. To this end, the review begins by exploring the most common complications that arise following an ICU stay, with a focus on PICS and its related conditions. It then delves into additional health issues frequently observed in ICU survivors—such as chronic infections, procedure-related complications, or the exacerbation of pre-existing conditions—which, while not traditionally included in the definition of PICS, contribute significantly to long-term morbidity. Further, the review outlines key considerations for general practitioners, emphasizing the importance of adopting a structured and systematic approach to managing individuals with a history of ICU hospitalization. By addressing the complex and multifaceted nature of post-ICU recovery, this review seeks to strengthen the role of primary care physicians in ensuring continuity of care, optimizing treatment pathways, and ultimately improving long-term outcomes for ICU survivors.

**Table 1:** Post-ICU sequelae that are included in the extended PICS definition.

Category		References
**Physical**	**Neuromuscular** Muscle weakness Fatigue and reduced endurance Joint and nerve pain Neuromyopathies Ventilator-induced diaphragm weakness Frailty Chronic pain Joint contractures and ectopic ossifications Speech difficulties (related to neuromuscular impairment)	Herridge et al., 2023 [28] Appleton et al., 2015 [29] Koch et al., 2014 [30] Clavet et al., 2015 [31] Latronico et al., 2023 [32] Amacher et al., 2024 [33] Nakamura et al., 2021 [34] Skoretz et al., 2010 [35] Macht et al., 2011 [36] Schefold et al., 2017 [37]
	**Respiratory** Persistent breathlessness (dyspnea) Lung fibrosis (especially in ARDS patients) Chronic hypoxia requiring oxygen therapy Respiratory muscle weakness Tracheal stenosis (after prolonged intubation)	
	**Cardiovascular** Persistent tachycardia Stress-induced cardiomyopathy New-onset arrhythmias Autonomic dysfunction (e.g., postural hypotension) Increased risk of heart disease (heart failure, atherosclerosis) Increased risk of thrombosis (DVT, PE)	
	**Gastrointestinal and Metabolic System** Difficulty swallowing (dysphagia) Nutritional deficiencies and metabolic dysfunction Weight loss and sarcopenia New-onset diabetes and glucose dysregulation Chronic diarrhea and constipation (gut microbiome disruption, opioid use)	
	**Renal System** Chronic kidney disease Electrolyte imbalances Increased risk of hypertension	
	**Endocrine System** Adrenal insufficiency Hypothyroidism and non-thyroidal illness syndrome Hypogonadism (testosterone suppression, menstrual irregularities)	
	**Skin, Soft Tissue, and Wound Healing** Scarring caused by invasive procedures Procedure-related complications Oral injuries	
**Cognitive**	Memory deficits Difficulty with attention and concentration Impaired executive function (problem solving, decision making) Reduced processing speed Brain fog or confusion Speech difficulties Delayed cognitive recovery (especially in ARDS and sepsis survivors)	Calsavara et al., 2018 [38] Semmler et al., 2013 [39] Hopkins et al., 2005 [40] Pandharipande et al., 2013 [41] Iwashyna et al., 2010 [42]
**Psychological**	Anxiety Depression Post-traumatic stress disorder Sleep disturbances Emotional lability (mood swings) Panic attacks Paranoia Guilt Decreased libido Social withdrawal Loss of motivation Complicated grief (PICS-family)	Patel et al., 2016 [43] Jones et al., 2004 [44] Desai et al., 2011 [45] Griffiths et al., 2006 [46] Broomhead et al., 2002 [47] Wells et al., 1989 [48] Zatzick et al., 2008 [49] Dowdy et al., 2008 [50] Bjørnøy et al., 2023 [51] Hatch et al., 2018 [52]

ICU, intensive care unit; PICS, post-intensive care syndrome; ARDS, adult respiratory distress syndrome; DVT, deep vein thrombosis; PE, pulmonary embolism.

## 2. Methods

This is a narrative review aiming to summarize key challenges and opportunities in post-ICU care, with a focus on the role of general practitioners (GPs). We conducted a targeted literature search using PubMed and Google Scholar between January 2010 and February 2024. Search terms included combinations of “post-intensive care syndrome”, “ICU recovery”, “general practitioner”, “primary care”, and “rehabilitation after critical illness.” Preference was given to original research, guidelines, and high-quality reviews relevant to adult ICU survivors transitioning to community care. Additional references were identified through manual screening of bibliographies. Articles were selected for inclusion based on clinical relevance, conceptual contribution, and applicability to general practice. The synthesis was structured around common post-ICU sequelae and domains of care coordination.

## 3. Post-Intensive Care Syndrome (PICS) and PICS Family

### 3.1. Definition and Scope of PICS

PICS is a condition characterized by new or worsening impairments in physical, cognitive, or mental health status that persist beyond the acute hospitalization phase following a critical illness or ICU admission [53]. It is increasingly recognized as a major consequence of modern critical care, affecting a substantial proportion of ICU survivors. Research suggests that more than half of ICU survivors exhibit at least one PICS-related symptom, often leading to long-term functional limitations and reduced quality of life [18,41]. The current definition of PICS excludes patients with primary neurological conditions, such as traumatic brain injuries and cerebrovascular accidents, even though these patients often experience similar or even more severe post-ICU impairments.

Risk factors for PICS are not universally defined and vary across studies. However, they are generally categorized into the following two broad groups: 1. Pre-existing patient-related factors, such as comorbidities, age, baseline frailty, and pre-hospital functional status. 2. ICU-related factors, including delirium, prolonged sedation, mechanical ventilation, sepsis, multi-organ dysfunction, and exposure to invasive procedures [54,55]. Table 2 summarizes the most frequently reported risk factors for PICS. The relative contribution of individual risk factors to PICS varies by domain. For instance, delirium and prolonged sedation have been consistently linked to long-term cognitive impairment [56], while prolonged immobility, sepsis, and neuromuscular blockade are more strongly associated with physical dysfunction [57]. Pre-existing conditions, such as frailty, cognitive decline, or mental health disorders, can increase vulnerability but are not always predictive of specific PICS outcomes [41]. While the formal weighting of these factors is not yet standardized, ICU-related exposures, especially delirium, tend to show stronger and more consistent associations with long-term sequelae across multiple studies [58].

**Table 2:** PICS Risk Factors.

PICS Component	Modifiable Risk Factors	Non-Modifiable Risk Factors	References
PICS (General)	Prolonged ICU stay, hypoglycemia, hypoxemia, inadequate communication with ICU staff, restricted visitation policies.	Advanced age, pre-existing medical conditions, previous ICU admission, history of anxiety or depression requiring medication.	Needham et al., 2012 [59] Rawal et al., 2017 [60] Inoue et al., 2019 [58] Lee et al., 2020 [61]
Physical	Prolonged immobility, bed rest, catabolic state, microvascular ischemia, extended mechanical ventilation, hyperglycemia, use of glucocorticoids and neuromuscular blocking agents, sleep disturbances.	Acute respiratory distress dyndrome (ARDS), advanced age, hyperoxia, vasopressor administration.	Hopkins et al., 1999 [62] Lee et al., 2020 [61] Fan et al., 2014 [63] Stevens et al., 2007 [64]
Cognitive	ICU delirium, prolonged mechanical ventilation, hypoxia, dysglycemia, use of psychotropic medications, blood transfusions, blood pressure fluctuations.	Advanced age, comorbidities, lower education level, pre-existing cognitive impairment (e.g., dementia), presence of APOE allele, severity of illness.	Pandharipande et al., 2013 [41] Jackson et al., 2010 [65] Girard et al., 2010 [56] Iwashyna et al., 2010 [42]
Psychological	Duration of ICU delirium, distressing ICU memories, prolonged sedation, opioid and benzodiazepine dosage, nightmares, breathlessness, alcohol consumption.	Female sex, pre-existing depressive symptoms, poor pre-ICU physical functioning, lower education level, history of anxiety or depression.	Lee et al., 2020 [61] Parker et al., 2015 [66]
PICS-F (Family-Related)	Poor communication with ICU staff, restricted visitation, financial stress, inadequate caregiver education, lack of support systems.	Advanced age, prolonged ICU stay of a loved one, prior experience with ICU care, history of anxiety or depression requiring medication.	Cameron et al., 2016 [67] Shirasaki et al., 2024 [68]

ICU, intensive care unit; APOE, apolipoprotein E.

### 3.2. Physical Impairments in PICS

Physical dysfunction is a key component of PICS, affecting approximately one-third of ICU survivors. One of the hallmarks of PICS-related physical impairment is ICU-acquired muscle weakness (ICUAW), which affects nearly 40% of ICU survivors [29]. This condition results from immobility, systemic inflammation, critical illness polyneuropathy, and corticosteroid use, leading to profound muscle atrophy. In many cases, motor deficits persist for months or even years post-discharge [30]. Joint contractures and ectopic ossifications may lead to additional mobility restrictions [31]. Other common manifestations include fatigue, sleep disturbances, weight loss, respiratory dysfunction, and dysphagia [32]. These impairments often result in reduced mobility, dependence on caregivers, and difficulty in resuming daily activities, such as cooking, walking, or medication management [33]. Respiratory muscle weakness increases the risk of dyspnea and long-term ventilatory dependence. Muscle weakness is also closely associated with cognitive and psychological impairments, highlighting the complex interplay of PICS symptoms [34]. However, despite its significant impact on post-ICU recovery, no standardized therapeutic intervention exists for ICUAW [69].

Post-extubation dysphagia is an increasingly recognized physical impairment following critical illness, particularly among patients who have undergone prolonged mechanical ventilation or had neurological involvement. It is associated with aspiration, pneumonia, malnutrition, and prolonged hospitalization, and it often persists long after ICU discharge. Studies suggest that up to 62% of ICU survivors experience some degree of dysphagia in the early post-ICU period, with a significant proportion requiring ongoing speech and swallowing therapy. Despite its high prevalence and impact, dysphagia is frequently underdiagnosed in both ICU and post-ICU settings. Incorporating systematic screening and early rehabilitation is essential for improving long-term outcomes and quality of life in this population [35,36,37].

### 3.3. Cognitive Dysfunction in PICS

Cognitive impairments range from mild memory deficits to severe executive dysfunction. Affected individuals may experience memory loss and poor concentration, slowed cognitive processing speed, speech difficulties, and, finally, challenges with executive functioning, including planning, organization, and problem solving. While many survivors experience gradual cognitive improvement within the first year, others, particularly those recovering from ARDS or septic shock, may have persistent cognitive dysfunction that impairs their ability to return to work or manage daily responsibilities [38,39,40]. Among the strongest ICU-related predictors is delirium, with multiple studies demonstrating a clear association between delirium duration and long-term cognitive decline [41,56]. Other contributing factors include prolonged mechanical ventilation, deep sedation, hypoxia, blood pressure fluctuations, and exposure to psychoactive medications. Additionally, pre-existing cognitive impairment, advanced age, lower educational attainment, and greater severity of illness increase vulnerability to cognitive PICS, though they may not independently predict its development [42].

### 3.4. Psychological and Emotional Distress in PICS

Psychological disturbances are frequently reported in PICS, significantly reducing survivors’ mental well-being and social reintegration [43,44]. Commonly reported symptoms include depression and anxiety, panic attacks, PTSD, paranoia, guilt, decreased libido, and social withdrawal. These conditions often manifest alongside chronic fatigue, loss of motivation, and sleep disturbances, further complicating recovery [45,46,47]. Depression is a significant concern, as it is linked to prolonged absence from work and an increased risk of suicide [48,49]. Research suggests that 25% to 60% of ICU patients develop depression post-discharge [50,51]. PTSD is associated with exposure to a life-threatening event and is characterized by intrusive thoughts, avoidance behaviors, irritability, and paranoia. The prevalence of PTSD among ICU survivors varies widely, with estimates ranging from 4% to 62%. One systematic review found that 24% of ICU survivors exhibited PTSD symptoms for up to eight years post-discharge. Anxiety, though less extensively studied, is often linked to distressing ICU memories, hypervigilance, and persistent agitation, with prevalence rates ranging from 16% to 62% [22,52].

### 3.5. PICS-F (PICS Family)

PICS not only affects ICU survivors but also has a profound impact on their families and caregivers. The term PICS-F refers to the new-onset psychological symptoms experienced by family members following a loved one’s ICU stay [68]. These individuals frequently suffer from anxiety, depression, post-traumatic stress disorder (PTSD), and complicated grief, with symptoms that may persist for months or even years after the ICU experience.

Prevalence rates vary widely, largely due to differences in study designs and assessment methods. However, anxiety is particularly prevalent, affecting nearly half of family members within the first six months after ICU discharge [67,70,71]. Most documented PICS-F symptoms fall within the psychological domain, while somatic and physical manifestations remain underexplored. Though less well described in the literature, issues such as musculoskeletal discomfort and persistent fatigue have been reported and warrant further investigation to fully understand the broader impact of PICS-F on caregivers’ well-being. A comprehensive review highlighted that physical symptoms, such as fatigue, are significant yet often underreported components of PICS-F [68]. One study found that nearly half of the families of critically ill patients experienced fatigue during the ICU stay, with these symptoms persisting up to four months after discharge. Additionally, more than half of the family members reported poor sleep quality both during the ICU stay and at four months post-admission [72,73]. Notably, these physical symptoms were correlated with psychological symptoms, suggesting a complex interplay between physical and mental health in PICS-F, as also described in PICS.

## 4. ICU-Specific Complications Beyond PICS

In addition to PICS, many ICU survivors experience complications that are not strictly classified under PICS but still significantly impact long-term health. These complications can be isolated issues or may overlap with symptoms of PICS, exacerbating patient outcomes. Some of these ICU-specific complications can develop due to prolonged immobility, invasive procedures, mechanical ventilation, and systemic inflammation. Table 1 also includes a comprehensive summary of these complications.

### 4.1. Cardiovascular Complications

Cardiovascular issues are highly prevalent among ICU survivors and may persist long after discharge. These complications often arise due to sepsis, prolonged immobilization, mechanical ventilation, and systemic inflammatory responses [74,75]. Common post-ICU cardiovascular complications are as follows: 1. Myocardial dysfunction, stress-induced cardiomyopathy, left ventricular dysfunction, and myocardial ischemia. 2. Persistent tachycardia and new-onset arrhythmias, with increased risk of atrial fibrillation, ventricular ectopy, and conduction abnormalities. 3. Autonomic dysfunction and postural hypotension, leading to dizziness, syncope, and orthostatic intolerance. 4. Long-term cardiovascular disease risk. ICU survivors, particularly those recovering from sepsis and ARDS, have an elevated risk of developing heart failure and atherosclerosis [76,77,78].

### 4.2. Respiratory Complications

Respiratory dysfunction is common among ICU survivors, even in those with no prior history of lung disease. Patients requiring mechanical ventilation are at increased risk of persistent lung injury and ventilator-associated complications. Common post-ICU respiratory sequelae include the following: 1. Persistent breathlessness, often due to residual lung injury, diaphragm weakness, and impaired pulmonary function. 2. Post-ARDS lung fibrosis. ICU survivors of ARDS may develop restrictive lung disease and fibrotic lung changes; although, most exhibit gradual improvement over time. 3. Chronic hypoxia. A subset of patients, mostly with pre-existing lung disease, may require long-term oxygen therapy to maintain adequate oxygenation. 4. Ventilator-induced diaphragm dysfunction, leading to exercise intolerance and chronic respiratory failure [79].

### 4.3. Gastrointestinal and Metabolic Complications

Gastrointestinal and metabolic dysfunction are frequently observed in post-ICU patients, with consequences ranging from nutritional deficiencies to long-term endocrine imbalances. Dysphagia (swallowing dysfunction), resulting from prolonged intubation, increases the risk of aspiration pneumonia and malnutrition, and is commonly included in the core symptoms of PICS. Except for dysphagia [80], other gastrointestinal complications are as follows: 1. Chronic diarrhea and constipation, frequently due to prolonged antibiotic use, gut microbiome dysbiosis, and opioid-induced bowel dysfunction 2. Nutritional deficiencies and sarcopenia. ICU survivors frequently experience persistent weight loss, muscle wasting, and vitamin/mineral deficiencies, requiring nutritional rehabilitation.

### 4.4. Renal Complications

Acute kidney injury (AKI) is a common complication of ICU admission and may have significant long-term consequences. While the majority of patients recover from AKI, a substantial subset develops chronic kidney disease (CKD), necessitating lifelong monitoring and nephrology follow-up. Common post-ICU renal complications are as follows: 1. CKD, with some ICU survivors requiring long-term renal support due to impaired kidney function following AKI. 2. Electrolyte imbalances. ICU-related AKI can lead to chronic disturbances in sodium, potassium, and phosphate homeostasis. 3. Increased risk of hypertension and cardiovascular disease. CKD patients have a higher likelihood of developing cardiovascular complications post-ICU. Kidney function monitoring and electrolyte assessments prevent long-term renal deterioration in ICU survivors [81,82].

### 4.5. Endocrine Dysfunction

Endocrine disturbances are often underrecognized, despite affecting multiple hormonal pathways. Critical illness disrupts hypothalamic–pituitary–adrenal (HPA) axis function, leading to persistent metabolic and hormonal imbalances. These complications include the following: 1. Adrenal insufficiency resulting from prolonged steroid use, stress-induced adrenal suppression, or sepsis. 2. Hypothyroidism and non-thyroidal illness syndrome contributing to fatigue and metabolic slowing. 3. Hypogonadism, testosterone suppression in men, and menstrual irregularities in women affecting energy levels and overall recovery [28,81]. 3. New-onset diabetes and glucose dysregulation [83]. Many ICU survivors develop stress-induced hyperglycemia, which may progress to insulin resistance and long-term metabolic dysfunction [81,84].

### 4.6. Hematologic and Immunologic Complications

Patients may be left with persistent immune dysfunction and hematologic abnormalities, which can prolong recovery and increase susceptibility to infections and thrombotic events.

Common complications include the following: 1. Anemia, frequently due to chronic inflammation, critical illness-associated blood loss, or nutritional deficiencies, leading to fatigue and reduced exercise tolerance. 2. Hypercoagulability and increased risk of thrombosis. Particularly those recovering from sepsis, COVID-19, or prolonged immobility are at an elevated risk of deep vein thrombosis (DVT) and pulmonary embolism (PE). 3. Chronic immune dysfunction and ICU-related immunosuppression, which can increase susceptibility to recurrent infections and delay recovery [28,81,85].

### 4.7. Overall Increased Long-Term Mortality

Despite a decline in in-hospital mortality, ICU survivors, particularly patients treated for sepsis, have significantly elevated long-term mortality. Nearly one-third of sepsis survivors die within a year after discharge, and the risk remains elevated for several years [27,57]. The causes of this excess mortality are multifactorial. Residual organ damage, especially renal and cardiovascular dysfunction, and impaired immune responses contribute to increased susceptibility to reinfection, the exacerbation of chronic diseases, and cardiovascular events [27,28]. Sepsis often accelerates the trajectory of pre-existing comorbidities. It induces persistent inflammation and immunosuppression, known as the PICS-related persistent inflammation–immunosuppression–catabolism syndrome (PICS-PICS), which predisposes survivors to a downward spiral of physical and cognitive decline. Frailty, ICU-acquired weakness, and impaired functional recovery further compromise independence and increase health care utilization, contributing indirectly to mortality. These findings highlight the importance of comprehensive post-ICU follow-up and tailored rehabilitation strategies to mitigate long-term consequences.

### 4.8. Socioeconomic Sequelae and Return to Work

Beyond clinical impairments, ICU survivors often face profound socioeconomic consequences. Returning to work is delayed or impaired in a substantial proportion of survivors, particularly among those with persistent cognitive, psychological, or physical limitations. Studies have shown that only 40–60% of working-age ICU survivors resume employment within one year, and many experience reduced income or a shift in occupational roles. A recent meta-analysis of 52 studies found that only 36% of previously employed patients had resumed work by 1–3 months post-discharge, rising to 60% at one year. Among those who returned, 22–58% changed occupations or reduced hours, and many suffered long-term earnings losses [57,86]. Caregiver burden, out-of-pocket costs, and reduced social functioning further deepen the socioeconomic impact. Recognizing these socioeconomic complications is essential for general practitioners to tailor support, initiate vocational rehabilitation referrals, and guide conversations about expectations and recovery trajectories.

## 5. Complications Related to Invasive Procedures

Invasive procedures are central to ICU management, yet they carry risks of both early and delayed complications. Many such complications may manifest after discharge and require recognition and management by primary care professionals. Table 3 summarizes these complications and the suggested diagnostic and treatment options.

**Table 3:** Complications related to invasive procedures in ICU.

Procedure	Complication	Clinical Presentation	Diagnostic Tools	Management	References
Intubation/Tracheostomy	Tracheal Stenosis	Delayed cough, secretion retention, dyspnea, stridor, wheezing	CT scan, laryngotracheoscopy, bronchoscopy	Laser resection, balloon dilation, stenting, surgical resection	Bello et al., 2016 [87] Zouket al., 2021 [88]
	Tracheomalacia	Expiratory wheeze, secretion retention, cough	Dynamic CT, bronchoscopy, spirometry	Humidification, physiotherapy, CPAP, stenting, surgery	
	Tracheoesophageal Fistula (TEF)	Aspiration, cough with swallowing, fever, increased secretions	Chest X-ray, CT, barium swallow, endoscopy	Endoscopic stenting, sealing agents, surgical repair	Kim et al., 2020 [89] Dhiwakar et al., 2020 [90]
	Tracheostomy-Related Hemorrhage	Bleeding from tracheostomy, sentinel bleeding, massive hemorrhage	Clinical diagnosis	Emergency intubation, overinflation of cuff, surgery	Gilbey 2012 [91]
	Local Infection/Tracheocutaneous Fistula	Redness, discharge, persistent opening at stoma site	Clinical evaluation	Local wound care, antibiotics, surgical closure if needed	Jarosz et al., 2017 [92]
Central Venous Catheters (CVC/PICC)	Catheter-Related Thrombosis	Limb swelling, pain, venous congestion	Doppler ultrasound, CT venography	Anticoagulation, catheter removal, thrombolysis in selected cases	Rajasekhar et al., 2017 [93] Evans et al., 2018 [94] Kucher 2011 [95]
	Central Venous Stenosis	Edema, pain, superior vena cava syndrome, facial swelling, rare bleeding complications	CT/MR venography, contrast venography	Angioplasty, stenting, surgical bypass (rare)	Hussein et al., 2008 [96] Sonavane et al., 2015 [97]
	Post-Thrombotic Syndrome (PTS)	Chronic limb pain, heaviness, edema, skin changes, ulcers	Duplex ultrasound, clinical assessment	Compression therapy, anticoagulation, thrombolysis, lifestyle modification	Kahn 2016 [98] Tie et al., 2015 [99] Strijkers et al., 2017 [100]
ECMO (VA/Peripheral Cannulation)	Arterial Ischemia/Stenosis	Limb ischemia, claudication, PAD exacerbation	ABI measurement, duplex ultrasound, wound inspection	Vascular surgery consult, endovascular intervention, PAD treatment	Banks et al., 2024 [101] Shin et al., 2024 [102]
	Infection/Delayed Bleeding	Wound infection, signs of sepsis, bleeding at cannulation site	Clinical evaluation, imaging if abscess suspected	Antibiotics, wound care, surgical debridement	Banks et al., 2024 [101]

CT, computerized tomography; CPAP, continuous positive airway pressure; MR, magnetic resonance; ECMO, extracorporeal membrane oxygenation; VA, venoarterial; PAD, peripheral arterial disease; PICC, peripherally inserted central catheter.

### 5.1. Airway Complications

Tracheal stenosis is a common late complication following prolonged endotracheal intubation or tracheostomy. Iatrogenic injury is the leading cause of benign tracheal stenosis [103]. Intubation and hypotension contribute to mucosal ischemia and lead to granulation and fibrosis, narrowing the airway [87]. Risk is increased in patients with obesity, diabetes, autoimmune conditions, chronic respiratory inflammation female sex, or pregnancy [103,104,105]. Symptoms often emerge weeks or months post-extubation, as the tracheal lumen narrows by >50%. Early signs include cough and difficulty clearing secretions; advanced narrowing (<10 mm or <5 mm in diameter) may cause exertional dyspnea, stridor, or wheezing, often misdiagnosed as asthma or bronchitis [87,106,107]. Diagnosis is best achieved through fiberoptic laryngotracheoscopy or bronchoscopy, though imaging, X-ray, computerized tomography (CT), and magnetic resonance imaging (MRI) can assist. Treatment includes laser resection, balloon dilation, stenting, or surgical resection, guided by a multidisciplinary team [87,88].

Tracheomalacia involves the dynamic collapse of the trachea, especially during forced expiration or coughing. It results from prolonged intubation or tracheostomy and presents similarly to tracheal stenosis. Diagnosis is made via dynamic CT or bronchoscopy, showing >50% expiratory narrowing. Spirometry may indicate obstruction but is non-diagnostic [87,108,109]. Management ranges from humidification and physiotherapy to continuous positive airway pressure (CPAP) ventilation, tracheal stenting, or surgery. A longer tracheostomy tube bypassing the malacic segment may suffice. Tracheoesophageal Fistula (TEF) arises from posterior tracheal wall injuries due to intubation, tracheostomy, or pressure from nasogastric tubes or overinflated cuffs. Symptoms are dysphagia, aspiration, fever, increased secretions, or cough on swallowing, and typically develop within days to week. Chest X-rays may show aspiration pneumonia. Diagnosis is confirmed with barium esophagography, CT, and endoscopic visualization. Treatment includes endoscopic stenting, sealing agents, or surgical repair, requiring a multidisciplinary approach [87,89,90].

Tracheostomy-related hemorrhage due to erosion of the innominate artery is rare but life-threatening. Sentinel bleeds may precede massive hemorrhage. Emergency airway protection, cuff overinflation, and surgical repair are vital. Prevention includes avoiding excessive cuff pressure, neck hyperextension, and unnecessary tube movement [91]. Finally, local infections and tracheocutaneous fistulas may require local care or surgical intervention [92].

### 5.2. Central Venous Access Complications

Central venous catheters (CVCs), including peripherally inserted central catheters (PICCs), are essential in ICU care but predispose to venous thrombosis, particularly in the upper extremities [93,94]. Risk factors include malignancy, renal failure, prior thrombosis, and prothrombotic states. Chronic complications include venous occlusion, stenosis, and post-thrombotic syndrome (PTS) [93,95]. Stenosis affects 20–40% of hemodialysis patients with a history of central catheter use. Often asymptomatic, symptoms appear once >50% narrowing occurs and include ipsilateral limb swelling, pain, and superior vena cava syndrome [96,110]. CT or MR venography assists in diagnosis; contrast venography remains the gold standard [97,111].

Post-thrombotic syndrome (PTS) is a chronic complication of deep vein thrombosis (DVT), more common with extensive or recurrent thromboses. Symptoms include pain, heaviness, edema, skin discoloration, and venous ulcers. While less frequent after catheter-related thromboses, PTS can emerge years later, particularly in older or obese patients [98,99,100].

### 5.3. Arterial Complications and ECMO-Related Sequelae

Late complications following ECMO, especially venoarterial (VA) ECMO, include infection, arterial stenosis, and ischemia of the cannulated limb. Pre-existing peripheral arterial disease (PAD) increases the risk [101,102]. Symptoms may include limb pain, discoloration, or non-healing wounds. Follow-up with the ankle–brachial index measurements, duplex ultrasound, and wound assessment is essential [101,112]. Illness severity during the ICU stay also correlates with vascular complications, potentially independent of the procedure itself [102].

## 6. Prevention and Treatment of ICU Complications and PICS

### 6.1. Preventive Strategies

A variety of strategies have been suggested to prevent PICS and mitigate long-term complications associated with critical illness. One of the most widely recognized frameworks is the “ABCDEFGH” bundle, which aims to reduce ICU patients’ risk of delirium, cognitive impairment, and physical dysfunction [113,114]. This bundle is shown in Table 4. Implementing this structured approach within ICU settings has been shown to reduce long-term complications and promote faster functional recovery. Several additional supportive interventions have been proposed to further reduce the incidence and severity of PICS. These include the following: 1. Preventing episodes of hypoglycemia and hypoxemia, which are associated with worse neurological and physical outcomes. 2. Facilitating patient communication using assistive technologies, such as cellphones, tablets, and picture boards, to reduce the psychological distress caused by mechanical ventilation and sedation. 3. Utilizing ICU diaries, which provide patients and their families with a record of their ICU stay. Studies suggest that ICU diaries help reduce post-discharge PTSD and anxiety symptoms by allowing patients to reconstruct their experiences [54,115,116]. 4. Establishing peer support groups, which create a structured support network for ICU survivors, enhancing their ability to adapt to post-ICU life.

**Table 4:** The “ABCDEFGH” bundle for PICS prevention.

Component	Description	Rationale
A: Assess, Prevent, and Manage Pain	Regular pain assessment and appropriate pain management strategies to enhance patient comfort and recovery.	Uncontrolled pain significantly increases delirium rates.
B. Spontaneous Awakening and Breathing Trials	Encouraging spontaneous awakening and breathing trials to reduce sedation duration and promote ventilator weaning.	“Sedation vacations” help limit delirium incidence, reduce mechanical ventilation duration, and shorten ICU stay.
C: Choice of Sedation and Analgesia	Using light sedation strategies and appropriate analgesia to minimize cognitive decline and delirium risk.	Benzodiazepines and excessive sedation increase delirium rates and prolong ICU stay.
D: Delirium: Assess, Prevent, and Manage	Routine delirium screening, implementation of non-pharmacological interventions, and management of contributing factors.	Delirium is associated with increased mortality, prolonged ICU and hospital stay, and long-term cognitive decline.
E: Early Mobility and Exercise	Promoting early mobilization, physical therapy, and rehabilitation to prevent muscle atrophy and improve functional outcomes.	Supported by RCTs; muscle weakness is linked to cognitive and psychological sequelae.
F: Family Engagement and Empowerment	Involving family members in the care process, educating them about the patient’s condition, and providing emotional support.	Reduces the incidence and severity of PICS-F.
G: Good Sleep Hygiene	Encouraging sleep-promoting practices, reducing nighttime disturbances, and optimizing circadian rhythms to enhance recovery.	Prevents delirium and helps reduce ICU length of stay.
H: Handout Materials and Follow-Up	Providing educational resources and ensuring post-discharge follow-up to monitor and manage PICS-related complications.	Reduces stress and anxiety, informs family members, and positively impacts PICS-F outcomes.

ICU, intensive care unit; PICS, post-intensive care syndrome; PICS-F, PICS family. Table adapted from Ely et al., 2017 [113].

Early mobilization during ICU stays has emerged as a cornerstone intervention for mitigating the long-term physical and cognitive sequelae of critical illness [117]. Initiating physiotherapy and active mobilization, even in mechanically ventilated patients, is feasible and safe and has been associated with shorter durations of delirium, reduced ICU-acquired weakness, and improved functional outcomes at discharge and beyond. Interdisciplinary collaboration and structured protocols can overcome barriers to implementation, even in patients with significant illness severity [118,119].

Delirium is also linked to increased mortality, long-term cognitive impairment, and reduced quality of life. Preventive strategies, such as regular reorientation, promoting sleep hygiene, minimizing sedation (especially benzodiazepines), and ensuring early mobilization, form the backbone of non-pharmacological delirium prevention. Structured approaches, like the ABCDEF bundle, have demonstrated effectiveness in reducing the incidence and duration of delirium, while also improving overall ICU outcomes [114,120,121].

### 6.2. Treatment Strategies for PICS and ICU-Related Complications

Despite dedicated efforts to prevent PICS, a substantial number of ICU survivors continue to experience long-term complications. Addressing these issues effectively requires multidisciplinary rehabilitation and a patient-centered approach to care. Post-ICU clinics have emerged as centralized hubs for addressing PICS-related challenges, offering structured support, individualized counseling, and education to help patients and families navigate recovery after ICU discharge. These clinics provide comprehensive counseling to help patients understand what to expect following ICU discharge. They also offer educational resources to patients and their families for managing ongoing symptoms, guidance on nutrition to combat malnutrition and weight loss, and promoting healthy sleep practices to address the common issue of disrupted sleep after ICU stays. However, evidence regarding the effectiveness of post-ICU clinics remains inconsistent. Studies report substantial heterogeneity across care models, and a recent meta-analysis highlights low to very low-certainty evidence regarding their impact on outcomes, such as quality of life, mental health, and return to work. Thus, while conceptually valuable and increasingly implemented, the role of post-ICU clinics in improving patient-centered outcomes requires further rigorous evaluation before widespread adoption [122]. Moreover, despite their potential benefits, such clinics remain limited in number, largely due to organizational challenges, insufficient resources, and workforce shortages [123,124].

Both pharmacological and non-pharmacological interventions to address the diverse and often complex symptoms associated with PICS. Psychiatric complications, such as depression, anxiety, and post-traumatic stress disorder are common among ICU survivors. While medications, like antidepressants, anxiolytics, and sleep aids, are frequently used in clinical practice, current evidence does not suggest that pharmacologic treatments have specific efficacy for PICS-related psychiatric conditions beyond their general use in the broader psychiatric population [125]. In contrast, non-pharmacological strategies, particularly cognitive behavioral therapy (CBT), have emerged as the cornerstone of mental health treatment in PICS and PICS-F. CBT has demonstrated effectiveness in managing symptoms of depression, anxiety, and PTSD and is increasingly accessible through digital platforms, such as smartphone applications and online services. This mode of delivery offers a practical and scalable solution, especially in settings with limited resources or during times of restricted in-person access, such as pandemics [126].

Given that PICS often affects multiple organ systems, rehabilitation should be multidisciplinary. Physical recovery can be supported through physiotherapy and occupational therapy, which aim to rebuild muscle strength, enhance endurance, and promote independence in daily living activities. For individuals recovering from ARDS, pulmonary rehabilitation, including breathing exercises and tailored oxygen therapy, can be instrumental in restoring respiratory function [127]. Additionally, patients suffering from cognitive impairments, such as memory loss or executive dysfunction, may benefit from neuropsychological interventions, including structured cognitive training programs, to aid in their cognitive recovery and improve overall quality of life [128].

Equally important is the enhancement of patient education initiatives. By equipping survivors and their caregivers with comprehensive information about PICS and the recovery process, these programs can empower individuals to take an active role in their rehabilitation and long-term health. The integration of digital health technologies also holds significant promise. Telemedicine platforms and mobile health applications can support remote monitoring, facilitate timely interventions, and offer psychological support, particularly beneficial for patients in underserved or rural areas [129]. Finally, developing and implementing standardized guidelines for screening, diagnosing, and managing PICS in primary care settings will be critical. Establishing clear protocols can help ensure early identification and appropriate intervention, ultimately improving outcomes for ICU survivors [129].

## 7. The Challenge of Post-ICU Follow-Up

**Figure 2:**
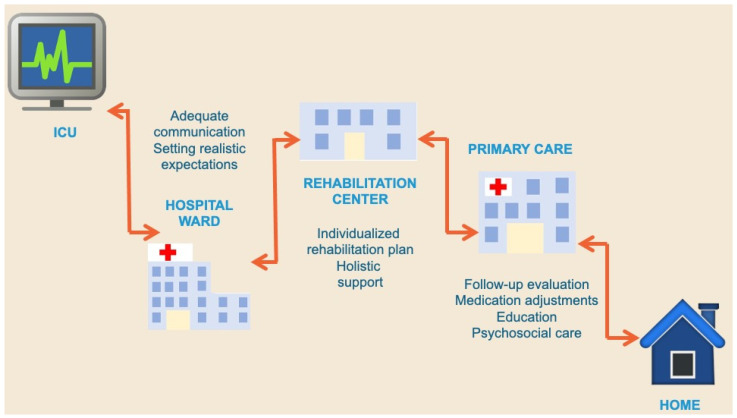
Pathways from ICU discharge to return home. This figure illustrates the various trajectories patients may follow after leaving the ICU. While some patients eventually return home, others require prolonged stays in rehabilitation or long-term care facilities, and some may never return home. The duration and sequence of each step vary between individuals. Direct discharge home from the ICU is uncommon. Information loss can occur at any transition point, and ICU or hospital readmissions are also possible along the pathway.

There is broad consensus that a substantial proportion of ICU survivors experience declining health following hospital discharge [28,62,130,131]. Increased healthcare utilization has been observed among these patients, underscoring their ongoing medical needs [127,132]. This trend highlights the urgent need for structured post-ICU monitoring, not only to manage PICS but also to identify and treat other post-ICU conditions that may not be strictly classified under PICS [25,133,134]. ICU survivors represent a particularly vulnerable patient population, often facing complex and underrecognized healthcare challenges after hospital discharge. Many patients struggle to navigate the healthcare system, encountering difficulties in accessing specialized care that addresses their unique post-ICU complications. The path from ICU to recovery significantly varies between patents, as shown in Figure 2. Research suggests that adherence to post-sepsis care protocols, including medication adjustments, screening for residual impairments, and monitoring for preventable health complications, can significantly improve long-term outcomes. However, studies indicate that only a small proportion of ICU survivors receive comprehensive follow-up care after hospital discharge [13].

ICUs are highly specialized settings defined by advanced technology, precise clinical interventions, and a concentrated focus on critical care medicine [135,136,137]. The clinical priorities and approach of ICU professionals often differ markedly from those in general medicine or community healthcare, where long-term management and holistic care are emphasized [138,139,140]. This divergence in focus can create communication gaps between ICU teams and outpatient providers, ultimately leading to fragmented care and poor transitions after discharge. A significant number of ICU survivors leave the hospital without a structured follow-up plan, increasing the risk of delayed recognition of complications and insufficient long-term support. Bridging these communication gaps is essential to improve care continuity and optimize recovery outcomes.

A qualitative study exploring the rehabilitation experiences of ICU survivors identified three interconnected elements as central to successful recovery [141]. First, knowledge and awareness are vital, as many patients report receiving little to no information about what to expect after discharge. This highlights the need for clear education as part of post-ICU care [142]. Second, regular evaluation and therapy are crucial, as structured assessments help detect persistent impairments and ensure timely intervention. Follow-up evaluations at regular intervals are particularly important for identifying late-onset complications, such as PTSD, which may not be immediately apparent but can significantly impact long-term recovery. Finally, personalized support, including psychosocial care and individualized rehabilitation plans, is fundamental to addressing the unique needs of each patient [143]. Despite the recognized importance of these components, they are rarely addressed during hospitalization. As a result, patients and their caregivers are often left unprepared for the physical, cognitive, and emotional challenges that may emerge after discharge [144].

## 8. The Role of the General Practitioner

In many regions, the responsibility for post-ICU care falls largely on GPs, who serve as the primary healthcare providers. Ideally, patients who have spent more than four days in intensive care should undergo a structured follow-up evaluation within 2–3 months of discharge [144]. Despite this recommendation, standardized screening protocols for PICS remain undefined, and the lack of a structured post-ICU care framework results in variability in follow-up care. Given the broad spectrum of PICS-related complications, conducting a comprehensive post-ICU assessment can be time-intensive and may exceed the scope of a routine GP consultation.

ICU follow-up clinics, where available, offer multidisciplinary assessments that cover both physical and psychological aspects of recovery. While these clinics do not replace primary care, they serve as referral hubs, directing ICU survivors to specialist services [23,145,146,147]. However, many regions lack such dedicated services, placing greater reliance on GPs to manage ICU survivors’ long-term needs [148,149]. GPs are, therefore, requested to identify residual symptoms associated with PICS. In addition to screening, GPs may be expected to coordinate referrals to relevant specialists. Patients experiencing ongoing issues may require continued care from physiotherapists, occupational therapists, mental health professionals, neurologists, or pulmonologists, depending on their specific needs. Another key aspect of post-ICU care involves the management of chronic conditions and new or exacerbated health problems, including diabetes, cardiovascular disease, or kidney dysfunction, all of which require long-term monitoring and tailored interventions.

GPs can also serve an important educational role by advising patients and their families on self-care strategies, lifestyle changes, and effective coping mechanisms to support long-term recovery. Finally, they can assess functional recovery by evaluating mobility, exercise tolerance, and the ability to perform daily activities. These assessments help determine whether additional rehabilitation services are necessary to support the patient’s ongoing recovery journey.

### 8.1. Barriers to Effective GP-Led Post-ICU Care

Despite their vital role in the long-term care of ICU survivors, GPs encounter several significant challenges that hinder their ability to provide comprehensive and effective follow-up care. One major obstacle is the limited access to clear and relevant ICU discharge summaries. While ICU teams typically generate detailed reports, these are often highly specialized, fragmented, or written with a focus on critical care terminology. As a result, GPs may struggle to extract the practical information needed to guide appropriate follow-up interventions in a primary care setting [150,151].

Another pressing issue is the absence of standardized guidelines for the screening and management of PICS. Table 5 shows the key post-ICU follow-up priorities to be considered in the outpatient setting. Without a universally accepted protocol, GPs are left to rely on their clinical judgment. This lack of structure increases variability in care and may lead to missed or delayed diagnoses [151]. Although there are no universally established post-ICU screening standards for clinical practice, recent consensus efforts have proposed core outcome domains for research involving survivors of acute respiratory failure, including ARDS. While these tools are primarily intended to facilitate consistency in research, they may also inform structured clinical follow-up, including in primary care, by highlighting key domains for monitoring ICU-related morbidity [152].

Time constraints during consultations further complicate the management of ICU survivors. A standard GP visit often does not allow sufficient time to conduct the in-depth evaluations necessary to identify and address the wide spectrum of issues associated with PICS. Without extended appointments or structured follow-up systems, important aspects of care may be overlooked.

In addition, many GPs have limited formal training in recognizing and managing ICU-specific complications. Although they are experienced in treating chronic and acute conditions across a wide population, GPs may not be familiar with the subtleties of post-ICU sequelae, such as neurocognitive impairments, lingering effects of sepsis, or complications from prolonged mechanical ventilation. This knowledge gap can further hinder the early identification and appropriate management of complex post-critical illness presentations [153,154]. Addressing these challenges requires improved communication between ICU and primary care teams, better educational resources for GPs, and the development of standardized care pathways tailored to the unique needs of ICU survivors.

**Table 5:** Summary of key post-ICU follow-up priorities.

System	Key Screening Tests and Referrals	When to Refer/Suggested Interventions	References
Cardiovascular	Blood pressure (sitting/standing) ECG (arrhythmias, QT prolongation) Echocardiogram (if signs of heart failure) Troponin (if chest pain or cardiac history)	Refer to cardiology if persistent arrhythmia, heart failure symptoms, or abnormal ECG findings Consider beta-blockers if autonomic dysfunction (e.g., POTS) is present	Prescott et al., 2014 [132] Schmidt et al., 2021 [155]
Respiratory	Dyspnea scale (e.g., modified Borg, mMRC) Pulmonary function tests Chest X-ray/CT (if persistent dyspnea, pulmonary fibrosis suspicion)	Refer to pulmonology if pulmonary function tests show restrictive/obstructive changes or if persistent hypoxia Prescribe pulmonary rehab for decreased exercise tolerance	Desai et al., 2011 [45] Vrettou et al., 2021 [22] Herridge et al., 2023 [28] Schmidt et al., 2021 [155]
Neuromuscular	Grip strength test Gait assessment (Timed Up and Go Test) 6-minute walk test Electromyography if persistent weakness	Refer to physiotherapy if significant muscle wasting or mobility impairment Consider nerve conduction studies for suspected critical illness polyneuropathy	Connolly et al., 2015 [156] Jolley et al., 2016 [157] Stevens et al., 2007 [64]
Endocrine	HbA1c (if ICU hyperglycemia or diabetes risk) Cortisol (morning sample if adrenal insufficiency suspected) Thyroid function (TSH, free T4)	Refer to endocrinology if adrenal or thyroid dysfunction Consider hydrocortisone trial for suspected adrenal insufficiency	Herridge et al., 2023 [28] Schmidt et al., 2021 [155]
Renal	Serum creatinine and eGFR (monitor for post-ICU AKI recovery) Electrolytes (K^+^, Na^+^, Mg^2+^)	Refer to nephrology if persistent eGFR <60 or electrolyte abnormalities	Herridge et al., 2023 [28] Schmidt et al., 2021 [155]
Gastrointestinal	Dysphagia screening test Nutritional status (BMI, albumin, pre-albumin) GI symptoms assessment (GERD, diarrhea)	Refer to speech therapy if dysphagia Prescribe proton pump inhibitors for stress-related GI issues	Herridge et al., 2023 [28] Schmidt et al., 2021 [155]
Hematologic	Complete blood count (check anemia, leukocytosis, thrombocytopenia) Coagulation panel (D-dimer, INR/PTT) Ferritin (if suspected iron deficiency)	Refer to hematology for unexplained cytopenias or hypercoagulability	Iba et al., 2020 [158] Schmidt et al., 2021 [155]
Infectious Risk (Asplenic)	Vaccination review (pneumococcal, meningococcal, Hib)	Provide missing vaccines and emergency ID/passport	Lefebvre et al. 2008 [159]
Cognitive	Montreal Cognitive Assessment (MoCA) or Mini-Mental State Exam (MMSE)	Refer to neuropsychology if cognitive impairment affects daily living, ocupational therapy, caregiver guidance	Hopkins et al. 2005 [40] Herridge et al. 2023 [28] Schmidt et al. 2021 [155]
Psychological	PTSD and depression screening (GAD-7, PHQ-9, IES-6)	Start antidepressants/CBT for persistent PTSD or depression	Bienvenu et al. 2012 [160] Hosey et al. Crit Care 2020 [161]
Functional	Frailty Index (Rockwood, Fried Frailty Criteria) Barthel Index (assess daily living independence) Physical rehab assessment	Refer to occupational therapy/rehab medicine if frailty or functional decline Consider home modifications for elderly ICU survivors	Griffiths et al. 2007 [162] Herridge et al. 2023 [28] Schmidt et al. 2021 [155]
Social	Caregiver burden assessment Work/financial hardship evaluation Social reintegration support	Connect with social workers for financial/workplace support Refer to peer support groups for ICU survivors	Schmidt et al. 2021 [155] Griffiths et al. 2007 [162] Kamdar et al. 2020 [86] Mantziou et al. 2022 [163]

ECG, electrocardiogram; POTS, postural orthostatic tachycardia syndrome; mMRC, modified medical research council scale; CT, computerized tomography; HbA1c, hemoglobin A1C; TSH, thyroid-stimulating hormone; eGFR, estimated glomerular filtration rate; AKI, acute kidney injury; GERD, gastroesophageal reflux disease; BMI, body mass index; GI, gastrointestinal; INR, international normalized ratio; PTT, partial thromboplastin time; PTSD, post-traumatic stress disorder; GAD-7, general anxiety disorder-7; PHQ-9, patient health questionnaire-9; IES-6, impact of event scale-6; CBT, cognitive behavioral therapy.

### 8.2. Strategies to Improve GP Involvement in Post-ICU Care

Improving the quality of post-ICU care requires the implementation of several coordinated strategies. One essential step is enhancing communication between ICU teams and primary care providers. Discharge summaries should be structured to emphasize the most relevant information for ongoing care, such as the patient’s functional status at discharge, current medications, and any specific follow-up recommendations [164,165]. In addition, fostering direct communication between intensivists and GPs, whether through handover calls or shared digital platforms, can help bridge knowledge gaps, clarify clinical priorities, and ensure smoother transitions in care.

**Figure 3:**
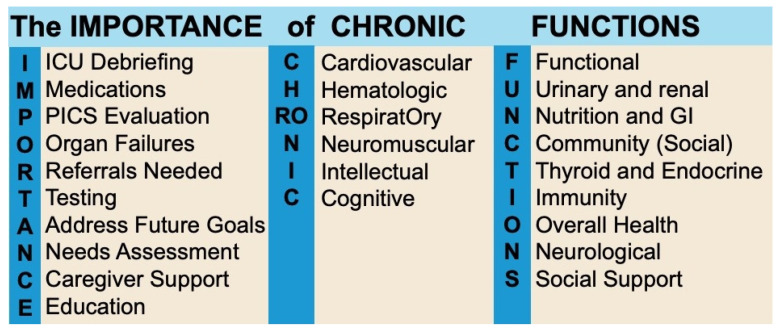
Suggested framework for post-ICU follow-up after hospital discharge. The left side illustrates the recommended approach for the first clinic visit, as proposed by Flores et al. (2024) [147], summarized by the mnemonic IMPORTANCE. This framework supports comprehensive recovery by addressing ICU debriefing, medication management, assessment for PICS (cognitive, psychiatric, and physical impairments), the resolution of organ failures, specialist referrals, follow-up testing, goals-of-care discussions, needs assessment, caregiver support, and education on recovery expectations. Not all elements are required at every visit, allowing for individualized care. The right side introduces the CHRONIC FUNCTIONS mnemonic, outlining key priorities for ongoing post-ICU follow-up, further detailed in Table 5.

Another critical area for improvement involves the development of standardized post-ICU screening protocols tailored for use in primary care. Integrating PICS-specific tools into routine GP follow-up visits would facilitate the early detection of complications and enable timely interventions. These protocols would serve as a practical framework for GPs navigating the complex recovery needs of ICU survivors. Table 5 shows some key post-ICU follow-up priorities that can be considered by GPs, while Figure 3 shows an approach to post-ICU clinic visits suggested by Flores et al. [147].

Once in primary care, routine follow-up can be guided by tools adapted for this setting. Screening instruments such as the Hospital Anxiety and Depression Scale (HADS), Montreal Cognitive Assessment—Blind (MoCA-Blind), PHQ-2, and 6-min walk test (6MWT) have been endorsed through international consensus for use in survivors of acute respiratory failure and PICS [152,155]. These tools may help GPs identify late-onset complications, including PTSD, depression, cognitive decline, and physical deconditioning. The concept of “functional reconciliation”—assessing changes from baseline in physical, cognitive, and psychological domains—has been proposed to structure outpatient evaluations. In this context, core outcome measures offer a promising path toward standardization and quality improvement in ICU survivorship care, even in the absence of universal post-ICU screening standards [152,155].

Telemedicine also presents a valuable opportunity to strengthen post-ICU care. Through virtual platforms, GPs can remotely monitor patients, conduct follow-up assessments, and provide psychological support—all of which are particularly beneficial for those with limited mobility or living in remote areas. Furthermore, virtual consultations can connect patients with peer support groups, fostering emotional resilience and reinforcing social support networks crucial for long-term recovery [13].

Finally, expanding educational opportunities for GPs is vital to equipping them with the knowledge and tools needed to manage the diverse range of post-ICU conditions. Targeted training programs focusing on PICS and related complications can provide practical clinical guidance. These may include workshops, online learning modules, and interdisciplinary case discussions, all aimed at enhancing awareness and competence in managing ICU survivorship within the primary care setting. Given the increasing relevance of post-ICU care in general practice, incorporating key aspects of ICU survivorship into GP training curricula may be a valuable consideration to better prepare future practitioners for this role [155].

## 9. Conclusions

The growing population of ICU survivors presents an evolving challenge for healthcare systems worldwide. While advancements in critical care medicine have improved survival rates, they have also led to an increase in patients facing long-term physical, cognitive, and psychological impairments known as PICS. In addition, a substantial proportion of ICU survivors develop complications beyond the scope of PICS, including cardiovascular, respiratory, metabolic, renal, and neuropsychiatric disorders, further complicating their recovery. Despite the significant burden of post-ICU morbidity, follow-up care remains fragmented and inconsistent. While dedicated ICU follow-up clinics exist in some healthcare systems, many ICU survivors rely on primary care providers for long-term management. However, gaps in communication between ICU teams and outpatient care providers, along with the absence of standardized post-ICU screening protocols, contribute to suboptimal recovery outcomes. To bridge these gaps, a multidisciplinary and proactive approach is necessary. ICU teams must ensure effective discharge planning, providing structured summaries with clear post-discharge recommendations. Primary care physicians, in turn, should be equipped with practical tools to identify and manage PICS-related complications, facilitate appropriate specialist referrals, and coordinate rehabilitation efforts. The integration of telemedicine, peer support networks, and digital health innovations further holds promise in enhancing access to follow-up care and psychological support. Moving forward, healthcare systems must prioritize education and training for primary care providers on post-ICU complications, develop standardized follow-up frameworks, and encourage greater collaboration between intensivists and GPs. Strengthening these aspects of post-ICU care will not only improve quality of life for ICU survivors but also reduce hospital readmissions, leading to better long-term health outcomes and more efficient healthcare utilization. Ultimately, post-ICU care should extend beyond survival to focus on recovery, reintegration, and long-term well-being. By addressing the current gaps in post-ICU follow-up, particularly at the primary care level, healthcare providers can ensure that ICU survivors receive the structured support they need to regain function, independence, and quality of life after critical illness.
